# *In vitro* activity of cefiderocol against *Pseudomonas aeruginosa* demonstrating evolved resistance to novel β-lactam/β-lactamase inhibitors

**DOI:** 10.1093/jacamr/dlad107

**Published:** 2023-10-03

**Authors:** Ryan K Shields, Ellen G Kline, Kevin M Squires, Daria Van Tyne, Yohei Doi

**Affiliations:** Department of Medicine, University of Pittsburgh, Pittsburgh, PA, USA; Center for Innovative Antimicrobial Therapy, University of Pittsburgh, Pittsburgh, PA, USA; Antibiotic Management Program, University of Pittsburgh Medical Center, Pittsburgh, PA, USA; Department of Medicine, University of Pittsburgh, Pittsburgh, PA, USA; Department of Medicine, University of Pittsburgh, Pittsburgh, PA, USA; Department of Medicine, University of Pittsburgh, Pittsburgh, PA, USA; Center for Innovative Antimicrobial Therapy, University of Pittsburgh, Pittsburgh, PA, USA; Center for Evolutionary Biology and Medicine, University of Pittsburgh, Pittsburgh, PA, USA; Department of Medicine, University of Pittsburgh, Pittsburgh, PA, USA; Center for Innovative Antimicrobial Therapy, University of Pittsburgh, Pittsburgh, PA, USA; Departments of Microbiology and Infectious Diseases, Fujita Health University School of Medicine, Toyoake, Aichi, Japan

## Abstract

**Background:**

Cefiderocol demonstrates excellent activity against MDR *Pseudomonas aeruginosa*; however, the activity against isolates from patients previously treated with β-lactam agents is unknown. We aimed to determine the activity of cefiderocol against *P. aeruginosa* collected before and after treatment with traditional β-lactams and new β-lactam/β-lactamase inhibitors.

**Methods:**

Cefiderocol MICs were determined in triplicate in iron-depleted cation-adjusted Mueller–Hinton broth and compared with β-lactam MICs tested by standard methods. All isolates underwent WGS analysis to identify mutations associated with resistance.

**Results:**

One hundred and seventy-eight *P. aeruginosa* isolates were evaluated; 48% (86/178) were non-susceptible to ceftazidime/avibactam, ceftolozane/tazobactam and/or imipenem/relebactam. The cefiderocol MIC_50_ and MIC_90_ were 0.12 and 1 mg/L, respectively. Median cefiderocol MICs did not vary against isolates classified as MDR, XDR, or those non-susceptible to ceftazidime/avibactam, ceftolozane/tazobactam and/or imipenem/relebactam when compared with non-MDR isolates. Against isolates collected from patients previously treated with ceftolozane/tazobactam, cefiderocol MICs were increased 4-fold compared with baseline. Cross-resistance to cefiderocol was identified in 21% (3/14) of patients who developed treatment-emergent resistance to ceftolozane/tazobactam. Overall, 6% (11/178) of isolates demonstrated cefiderocol MICs ≥2 mg/L, which were disproportionately collected from patients previously treated with ceftolozane/tazobactam (73%; 8/11). Isolates with reduced cefiderocol susceptibility harboured mutations in *ampC*, *tonB*-dependent receptors, the response regulator *pirR* and *ftsI*.

**Conclusions:**

Cefiderocol demonstrates excellent *in vitro* activity against *P. aeruginosa* isolates exposed to other novel β-lactam agents; however, some exceptions were identified. Cross-resistance between cefiderocol and ceftolozane/tazobactam was evident, but not with ceftazidime/avibactam or imipenem/relebactam. Reduced cefiderocol susceptibility was mediated by mutations in *ampC* and *tonB*-dependent receptors.

## Introduction

*Pseudomonas aeruginosa* is a leading cause of healthcare-associated infections in the USA, with a significant percentage of isolates classified as MDR.^1^ Novel β-lactam/β-lactamase inhibitor agents, including ceftazidime/avibactam, ceftolozane/tazobactam and imipenem/relebactam, have demonstrated high rates of *in vitro* activity; however, treatment-emergent resistance has been reported shortly after their introduction into clinical practice.^2–4^ Cefiderocol is unique when compared with the newest generation of β-lactam/β-lactamase inhibitor agents given that it uses iron transport systems to gain entry into the bacterial cell. The agent demonstrates stability against most known mechanisms of antibiotic resistance, including MBLs.^5^ In surveillance studies, cefiderocol demonstrates high rates of *in vitro* activity against MDR *P. aeruginosa*, including against isolates that are resistant to ceftazidime/avibactam, ceftolozane/tazobactam and/or imipenem/relebactam.^6–10^ Although encouraging, isolates included in these studies were not collected from patients previously treated with novel β-lactams, and differential resistance rates may be due to the presence of MBLs. In fact, underlying resistance mechanisms are often not well characterized in surveillance studies, particularly for *P. aeruginosa* where β-lactam resistance is mediated by multiple adaptive mechanisms.

Given the potential utility of cefiderocol for treatment of refractory MDR *P. aeruginosa* infections, we sought to determine its *in vitro* activity against genetically diverse clinical *P. aeruginosa* isolates and assess the impact of prior β-lactam treatment. We hypothesized that prior treatment and resistance to traditional β-lactam agents would not impact cefiderocol activity; however, the development of resistance to novel β-lactam/β-lactamase inhibitors would lead to reduced susceptibility. Thus, we selected *P. aeruginosa* isolates from patients at our centre not previously treated with β-lactams (*n* = 31), isolates from patients previously treated with traditional β-lactams (*n* = 69), and serial isolates from patients before and after treatment-emergent resistance to novel β-lactam/β-lactamase inhibitors (*n* = 78). We used WGS to identify underlying mechanisms of resistance and evaluate cross-resistance between cefiderocol and other agents.

## Materials and methods

One hundred and seventy-eight *P. aeruginosa* isolates were selected for investigation, including isolates from an ongoing prospective study of healthcare-associated infections.^11^ MDR and XDR isolates were defined by consensus criteria.^12^ Prior antibiotic exposure was defined as receipt of β-lactam treatment for ≥3 days within 90 days prior to the isolate collection date. For patients treated with novel β-lactam/β-lactamase inhibitor agents, serial isolates were included to compare baseline and post-exposure isolates from the same patient. This resulted in the inclusion of 78 isolates collected from patients before and after treatment with ceftolozane/tazobactam (*n* = 14 patients, 36 isolates), ceftazidime/avibactam (*n* = 4 patients, 13 isolates) and imipenem/relebactam (*n* = 5 patients, 29 isolates). None of the patients had received prior treatment with cefiderocol at the time of isolate collection. Clinical characteristics of patients treated with ceftolozane/tazobactam and imipenem/relebactam have been reported previously.^4,13^

MICs were determined in triplicate by broth microdilution (BMD) methods according to CLSI guidelines to identify the modal MIC.^14,15^ Imipenem (0.03–32 mg/L) was tested with and without relebactam (4 mg/L); meropenem (0.06–64 mg/L) was tested with and without vaborbactam (8 mg/L). Ceftolozane (0.06–64 mg/L) and piperacillin (0.05–512 mg/L) were both tested with 4 mg/L of tazobactam. Ceftazidime (0.25–256 mg/L) was tested with and without avibactam (4 mg/L). Aztreonam (0.12–128 mg/L) and cefepime (0.25–256 mg/L) were also tested for completeness. Cefiderocol panels (range 0.03–32 mg/mL) were provided by IHMA (Schaumberg, IL, USA) and made onsite using Chelex^®^-treated, iron-depleted, cation-adjusted Mueller–Hinton broth.^15^ Quality control (QC) strains *P. aeruginosa* ATCC 27853, *Klebsiella pneumoniae* ATCC 700603, *Escherichia coli* ATCC 25922 and *K. pneumoniae* BAA-1705 were used throughout, and results were only included when QC strains were within CLSI reference ranges. All MICs were interpreted according to CLSI interpretive criteria.^15^

WGS was performed and analysed as previously described.^4,11,13^ Briefly, DNA was sequenced on the Illumina platform, assembled using SPAdes 3.14 and annotated with Prokka.^16,17^ SNPs were called using SAMtools, and MLST was performed with MLST v2.19 (https://github.com/tseemann/mlst).^18^ The presence or absence of β-lactamase genes was confirmed using ResFinder.^19^ Following a literature search, 19 genes of interest were chosen *a priori* for analysis (Table S1, available as Supplementary data at *JAC-AMR* Online). Protein sequences were compared with *P. aeruginosa* PAO1. Genomes are available under NCBI BioProjects PRJNA475751, PRJNA715186, PRJNA782612 and PRJNA872132.

Categorical and continuous variables were analysed using a chi-squared (or Fisher’s exact) test and Mann–Whitney U test, respectively. A two-tailed *P* value ≤0.05 was considered statistically significant.

## Results

Across 178 *P. aeruginosa* clinical isolates included in the study, 70 STs were represented; ST244 (*n* = 14) and ST245 (*n* = 11) were most common (Table S2). Twenty-two isolates were identified as high-risk clones by ST, including ST244 (*n* = 14), ST111 (*n* = 8) and ST235 (*n* = 2).^20^ Chromosomal *bla*_OXA-50-like_ genes were identified in 177 isolates; other β-lactamase genes identified included *bla*_CARB-2_ (*n* = 7), *bla*_OXA-9_ (*n* = 6), and *bla*_IMP-8_, *bla*_VIM-1_ and *bla*_OXA-10_ (*n* = 1 each). All isolates encoded chromosomal *ampC* genes [*Pseudomonas*-derived cephalosporinase (PDC)]. Thirty-two PDC variants were identified; PDC-338 (*n* = 13), PDC-382 (*n* = 10), PDC-51 (*n* = 8) and PDC-380 (*n* = 6) were most common. Eighty-five percent (152/178) of isolates were classified as MDR (*n* = 50) or XDR (*n* = 102).^12^

Among prospectively collected epidemiological isolates (*n* = 100), rates of susceptibility to aztreonam, ceftazidime, cefepime, imipenem and piperacillin/tazobactam were 17%, 36%, 35%, 55% and 29%, respectively (Table S2). Rates of susceptibility to meropenem and meropenem/vaborbactam were both 58%. By comparison, susceptibility rates for ceftazidime/avibactam, ceftolozane/tazobactam and imipenem/relebactam were 85%, 83% and 83%, respectively. Ninety-four percent of isolates demonstrated cefiderocol MICs <2 mg/L. The cefiderocol MIC_50_ and MIC_90_ were 0.06 and 0.25 mg/L, respectively. Median cefiderocol MICs did not vary against MDR compared with XDR isolates (0.06 versus 0.12 mg/L; *P = *0.27). Median cefiderocol MICs against isolates non-susceptible to ceftazidime/avibactam, ceftolozane/tazobactam and/or imipenem/relebactam were 0.06, 0.03 and 0.06 mg/L, respectively. Sixty-nine percent (69/100) of isolates were collected from patients previously exposed to β-lactams within 90 days preceding isolate collection. Median (range) days of prior exposure for patients who received cefepime (*n* = 25), meropenem (*n* = 27) and piperacillin/tazobactam (*n* = 49) were 8 (3–19), 11 (3–50) and 8 (3–39), respectively. Cefiderocol MICs did not vary among patients who did (*n* = 69) or did not (*n* = 31) receive prior β-lactams (median = 0.06 mg/L for both). Only six isolates were collected from patients previously exposed to ceftazidime/avibactam or ceftolozane/tazobactam (*n* = 3 each), and none were collected from patients previously exposed to imipenem/relebactam.

To further explore the impact of treatment-emergent resistance to these novel agents, cefiderocol susceptibility was evaluated against 36 isolates (15 baseline, 21 post-exposure) from 14 patients that developed treatment-emergent resistance to ceftolozane/tazobactam.^4^ Baseline isolates from 12 patients belonged to unique STs; the final two isolates each had a unique allele profile that was not identifiable within the MLST database. Five PDC variants were present at baseline; PDC-5 was most common (*n* = 6). All isolates carried *bla*_OXA-50-like_; however, no MBL or other serine β-lactamase genes known to confer ceftolozane/tazobactam resistance were found.^21,22^ Following ceftolozane/tazobactam treatment [median (range) duration = 19 (3–52) days], median ceftolozane/tazobactam MICs increased from 2 mg/L at baseline to 64 mg/L after exposure (*P *< 0.0001). Comparable MICs changes were noted for ceftazidime/avibactam (from 4 to 64 mg/L, *P *< 0.0001; Table 1). Treatment-emergent mutations in PDC were identified in 79% (11/14) of patients.^4^ Overall, median cefiderocol MICs increased from 0.12 mg/L among baseline isolates to 0.5 mg/L among isolates collected after ceftolozane/tazobactam exposure (*P *= 0.1034; Table 1). Fourteen percent (3/21) of post-exposure isolates yielded cefiderocol MICs >4 mg/L; each produced undefined variant PDC and harboured mutations in *pirR*, a regulator of the *tonB*-dependent receptor (TBDR) *pirA*. Two of the three resistant isolates also contained mutations in *ftsI*, which encodes penicillin-binding protein 3 (PBP3) in *P. aeruginosa*.

**Table 1.** Comparison of cefiderocol MIC values against isolates collected before and after treatment with ceftolozane/tazobactam, ceftazidime/avibactam or imipenem/relebactam

		Baseline			Post-exposure			
	Agent	MIC_50_ (mg/L)	MIC_90_ (mg/L)	MIC range (mg/L)	MIC_50_ (mg/L)	MIC_90_ (mg/L)	MIC range (mg/L)	*P* value^a^
		*n* = 15			*n* = 21			
C/T exposure	CZA	4	16	1–32	64	256	4–256	<0.0001
	C/T	2	4	0.25–8	64	256	1 to >64	<0.0001
	FDC	0.12	1	≤0.03–2	0.5	8	≤0.03–16	0.1034
	I-R	2	8	≤0.06–8	2	4	0.25–32	0.6692
	MVB	8	32	1–32	16	32	0.5–64	0.252
		*n* = 4			*n* = 9			
CZA exposure	CZA	4	8	1–8	16	64	4–128	0.0238
	C/T	2	2	1–2	4	16	2–16	0.0280
	FDC	0.06	0.12	≤0.03–0.12	0.06	0.25	≤0.03–0.25	>0.9999
	I-R	1	1	0.25–1	1	4	0.12–8	0.4965
	MVB	32	64	8–64	8	32	2–32	0.0909
		*n* = 12			*n* = 17			
I-R exposure	CZA	8	128	4–256	32	128	8–256	0.1986
	C/T	16	128	1 to >64	8	64	1 to >64	0.4307
	FDC	0.25	1	0.12–2	0.5	2	0.06–2	0.4038
	I-R	1	2	0.25–2	4	16	2–32	<0.0001
	MVB	8	16	2–32	16	64	8–64	0.0007

MIC_50_ and MIC_90_ values are rounded to the nearest doubling dilution. C/T, ceftolozane/tazobactam; CZA, ceftazidime/avibactam; FDC, cefiderocol; I-R, imipenem/relebactam; MVB, meropenem/vaborbactam.

a Comparison of non-rounded MIC_50_ by Mann–Whitney U test.

Next, 13 isolates (*n* = 4 baseline, *n* = 9 post-exposure) from four patients who developed treatment-emergent ceftazidime/avibactam resistance were analysed [median (range) duration of therapy = 10 (5–21) days]. Median ceftazidime/avibactam MICs increased from 4 mg/L at baseline to 16 mg/L following ceftazidime/avibactam exposures (*P *= 0.0238). Median ceftolozane/tazobactam MICs increased from 2 to 4 mg/L (*P *= 0.0280; Table 1). Post-exposure isolates in three of four patients showed mutations in both *ampD* and the *mexAB-oprM* operon, or its repressor gene *mexR*. Post-exposure isolates from the fourth patient demonstrated a mutation in *ftsI* (G63D). In addition, mutations were identified in PDC at amino acid positions 146 (Q146K) and 319 (P319A) among isolates collected from two patients. Median cefiderocol MICs did not change following treatment-emergent ceftazidime/avibactam resistance (both 0.06 mg/L, Table 1). Imipenem/relebactam and meropenem/vaborbactam median MICs did not significantly increase or decrease following ceftazidime/avibactam exposure (Table 1 and Table S2).

Against 29 isolates [*n* = 12 baseline, *n* = 17 post-exposure; median (range) duration = 14 (3–28) days] from five patients treated with imipenem/relebactam, the median cefiderocol MIC was 0.25 mg/L.^13^ Sixty percent (3/5) of patients treated with imipenem/relebactam had previously developed treatment-emergent resistance to ceftolozane/tazobactam (*n* = 2) or ceftazidime/avibactam (*n* = 1). The baseline isolate from each patient belonged to a unique ST and produced a unique PDC variant. Median imipenem/relebactam MICs increased significantly following treatment (from 1 to 4 mg/L, *P *< 0.0001), which was attributed to mutations in *mexAB-oprM* and *mexEF-oprN* efflux operons.^13^ Median meropenem/vaborbactam MICs increased in parallel from 8 to 16 mg/L (*P = *0.0007). By comparison, median cefiderocol MICs increased from 0.25 to 0.5 mg/L post-exposure, which was not statistically significant (Table 1). Two post-exposure isolates demonstrated cefiderocol MICs ≥2 mg/L, which were collected from a patient with prior treatment-emergent resistance to ceftazidime/avibactam or ceftolozane/tazobactam (one each).

**Figure 1.**
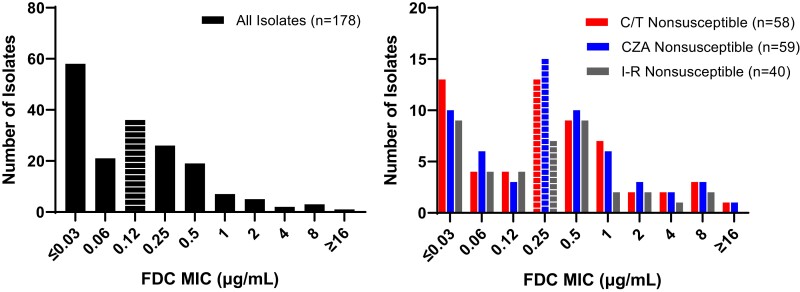
Overall distribution of cefiderocol MICs against all isolates (left) and those non-susceptible to ceftolozane/tazobactam, ceftazidime/avibactam or imipenem/relebactam (right). Note: MIC_50_ for each group is noted with horizontal white stripes. C/T, ceftolozane/tazobactam; CZA, ceftazidime/avibactam; FDC, cefiderocol; I-R, imipenem/relebactam.

Taken together across all 178 isolates, the cefiderocol MIC_50_ and MIC_90_ were 0.12 and 1 mg/L, respectively (Figure 1). Against isolates non-susceptible to ceftolozane/tazobactam (*n* = 58) or ceftazidime/avibactam (*n* = 59), the cefiderocol MIC_50_ and MIC_90_ was 0.25 and 4 mg/L, respectively. Against imipenem/relebactam non-susceptible isolates (*n* = 40), the cefiderocol MIC_50_ and MIC_90_ were 0.25 and 2 mg/L, respectively (Table S2). Median cefiderocol MICs were increased from 0.12 to 0.25 mg/L against isolates collected after exposure to any one of ceftazidime/avibactam, ceftolozane/tazobactam or imipenem/relebactam (*n* = 47) compared with baseline isolates from the same patients (*n* = 31; *P *= 0.1442).

Eleven isolates from 10 unique patients demonstrated cefiderocol MICs above the MIC_90_ (cefiderocol MIC ≥2 mg/L). To determine genetic factors associated with reduced cefiderocol susceptibility, we assessed mutations in relevant genes by comparing the protein sequence of each gene with the PAO1 sequence (Table S1).^9,23,24^ Mutations in *ampC*, AmpC regulators (*ampD*, *ampR*), *ftsI* or *oprD* were encountered in all 11 isolates. The inner membrane proteins *exbB* and *exbD* were each WT in 91% (10/11) of isolates (Table 2). Sixty-four percent (7/11) of isolates harboured *ftsI* mutations; however, the presence or absence of *ftsI* mutations alone did not impact cefiderocol MICs (median MIC = 0.25 mg/L for both groups). Two isolates contained major disruptions in either *tonB2* or *tonB3*. Three additional TBDR systems were analysed, including *fecA* (with *fecI* and *fecR*), *piuA* (or *piuD*, with *piuC*) and *pirA* (with *pirR* and *pirS*). Compared with the PAO1 *P. aeruginosa* reference sequence, mutations were identified in each TBDR system. Among serial isolates collected from the same patient, sequence changes were observed in *piuD* and *pirR,* but not *fecA*, *fecI*, *fecR*, *piuA*, *piuC or pirS.* In the *pirA* TBDR system that includes *pirR* and *pirS*, 54.5% (6/11) of isolates contained a single base pair insertion or deletion in a poly(dC) tract of *pirR* causing an early termination at amino acid position 201. Mutations in *pirA* were identified in all isolates and did not correlate with cefiderocol MICs. On the other hand, frameshift mutations in *pirR* were identified in 45% (5/11) isolates with reduced FDC susceptibility. Across isolates from patients treated with ceftolozane/tazobactam, ceftazidime/avibactam or imipenem/relebactam (*n* = 78 total), median cefiderocol MICs were 4 and 0.25 mg/L in isolates with or without a frameshift in *pirR* (*P *< 0.0001).

**Table 2.** Characterization of antibiotic MICs and relevant antibiotic resistance genes for isolates with cefiderocol MICs  ≥2 mg/L

Isolate		1393	2208	2230	1532	1423	1325	1653	1654	2772	2805	2524
Isolate type		Epidemiological	Epidemiological	Epidemiological	Baseline, C/T	Post C/T exposure	Post C/T exposure	Post C/T exposure	Post C/T exposure	Post I-R exposure	Post I-R exposure	Baseline, I-R
Patient					1	4	9	19	19	1	3	5
MIC (mg/L)	ATM	>128	32	16	32	32	128	>128	>128	64	64	64
	CAZ	512	32	128	64	128	128	>512	>512	128	16	512
	CZA	16	4	32	2	64	128	128	128	32	16	256
	C/T	>64	4	>64	2	64	128	128	128	8	2	128
	FDC	8	2	4	2	8	16	4	8	2	2	2
	FEP	>256	8	32	32	32	64	256	256	32	16	16
	IPM	32	8	2	32	0.5	1	32	32	32	16	0.5
	IPR	8	1	2	4	1	2	4	4	8	2	0.5
	MEM	64	4	8	8	2	16	64	64	16	32	4
	MVB	16	4	8	2	2	16	64	32	16	32	2
	TZP	>512	64	8	256	16	32	512	>512	512	64	64
ST^a^		348	3184	439	209	645	Undefined	796	796	244	164	151
PDC^a^		382	37	346	24	Undefined	Undefined	Undefined	Undefined	1	121	Undefined
*ampC*		R79Q, T105A, D234G, V251M	P7S, G27D, T105A, S131R, L145R, V205L, V356I, G391A	R79Q, T105A, G183D	T21A, G391A, T105A	**F147L**, L176H, **G183D**, E335K, T105A	R79Q, **G183D**, **243G ins**, **T105A**	**241R ins**, T105A	**241R ins**, T105A	WT	P7S, T105A	T105A, F147L, E247K, disrupted
*ampD*		WT	WT	G148A, S175L	R11L, G148A, D183Y	WT	G148A, S175L, **T168P (reversion)**	G148A, **G156R**, S175L	G148A, **G156R**, S175L	R11L, G148A, D183Y	G148A, D183Y	G148A, D183Y
*ampR*		D135N, R244W, G273E	E114A, I251V, G283E	3′ indel	WT	WT	WT	WT	WT	D135N, G283E, M288R	E114A, G283E, M288R	G283E, M288R
*ftsI*		R504L, P527S	D288A	WT	G216S	WT	**M460T**	**R504C**	**R504C**	WT	WT	T91A
*oprD* ^b^		Disrupted	Intact	Intact	Disrupted	Intact	Disrupted	**Intact**	**Intact**	Disrupted	Disrupted	Disrupted
*exbB1*		S223T	WT	WT	WT	WT	WT	WT	WT	WT	WT	WT
*exbD1*		WT	WT	WT	WT	WT	**I124V**	WT	WT	WT	WT	WT
*tonB1*		218insP, P230S	218insP, R259G, DP261-262AQ, E275Q, S286T, V295I, R302G, E307S	218insP	218insP	218insP	218insP	**WT**	**WT**	218insP, R259G, DP261-262AQ, E275Q, S286T, V295I, R302G, E307S	WT	R11C, 218insP
*tonB2*		WT	A172V	WT	WT	WT	WT	WT	WT	P94S	**Disrupted**	WT
*tonB3*		1nt ins AA7, fs	V144A	V144A	F57L, V144A	T81N, V144A	**S30P**, V144A	F57L, V144A	F57L, V144A	F57L, V144A	V144A	F57L, V144A
*fecI*		WT	WT	WT	L117V	WT	WT	WT	WT	WT	T102I	WT
*fecA*		A39T, V95A, H363R, W583R	V95A, V149G, T339A, GT358-359SA, R571Q	V95A, T339A, H363R, K627Q	H363R	V95A, H363R	T339A, H363R	T339A, H363R	T339A, H363R	T339A, H363R	T339A, H363R	V95A, T339A, H363R
*fecR*		A44T, H164Q, R208H	W99G, L101R, R208H, A230V, V247G, C270G	WT	WQR37-39LEC, H164Q	WQR37-39LEC, H164Q, R208H	D271A	G212D, P315S	G212D, P315S	P315S	WT	A230V, V306I
*piuA*		Q34H	Q34H	Q34H	—	Q34H	—	Q34H	Q34H	Q34H	Q34H	—
*piuD* ^c^		—	—	—	Q96K, V577A, S612T	—	**Q96K, V577A**	—	—	—	—	Q96K, V577A, K685T
*piuC*		G139A	R201H	V104I, G139A, R201H	V104I	V104I	V104I	WT	WT	WT	V104I	V14A
*pirS*		A328D	R77Q, S261P, A328D	G360D	WT	A328D, G360D	WT	G360D	G360D	WT	G360D	Contig break
*pirR*		1 bp del fs	F2L, G52A	1 bp del fs	1 bp del fs	G52A, reversion of fs	**1 bp ins fs**	**1 bp ins fs**	**1 bp ins fs**	WT	WT	WT
*pirA*		G23D, 1 nt del fs, 79*	A370T, E663A	A370T	G363S, A370T	A370T	A370T, **L642P**	A370T, E697K	A370T, E697K	A370T	A370T	Disrupted
*pvdS*		SYLF65-68del, Q69R	T155P	F47L, VTH180-182LAN	A119T, T155I, VTH180-182LAN, R186P	WT	**V43D**	T114P	T114P	WT	**WT**	VTH180-182LAN

Bold text denotes changes in post-exposure isolates compared with the baseline isolate from each patient. AA, amino acid; ATM, aztreonam; CAZ, ceftazidime; C/T, ceftolozane/tazobactam; CZA, ceftazidime/avibactam; del, deletion; FDC, cefiderocol; FEP, cefepime; fs, frameshift; I-R, imipenem/relebactam; ins, insertion; indel, insertion-deletion element; IPM, imipenem; MEM, meropenem; MVB, meropenem/vaborbactam; PDC, *Pseudomonas*-derived cephalosporinase; TZP, piperacillin/tazobactam.

a Undefined denotes ST or PDC variant that was not identified by MLST or ResFinder, respectively.

b Only major disruptions are reported.

c *piuD* is an orthologue of *piuA*.

## Discussion

Our data corroborate and extend prior surveillance studies describing the excellent *in vitro* activity of cefiderocol against *P. aeruginosa* clinical isolates. Overall, the cefiderocol MIC distribution we observed against clinical isolates from our centre was well aligned with prior reports.^6–9^ Against MDR and XDR isolates, the cefiderocol MIC_50_/MIC_90_ were 0.06/0.25 and 0.25/1 mg/L, respectively, confirming that commonly encountered mechanisms of antibiotic resistance in *P. aeruginosa* do not significantly contribute to reduced cefiderocol susceptibility. To extend these findings, we evaluated the impact of prior treatment with β-lactam antibiotics on the activity of cefiderocol. Not surprisingly, we found that exposure and resistance to traditional β-lactams did not impact the *in vitro* activity of cefiderocol. Next, we tested *P. aeruginosa* isolates recovered from patients previously treated with ceftazidime/avibactam, ceftolozane/tazobactam or imipenem/relebactam to represent cases where cefiderocol is likely to be considered as a treatment option. Against such isolates, the corresponding cefiderocol MIC_50_/MIC_90_ were 0.06/0.25, 0.25/4 and 0.25/2 mg/L, respectively. Median cefiderocol MICs were generally not impacted following exposure to any of these agents individually (Table 1); however, some exceptions were noted. Most importantly, median cefiderocol MICs were increased 4-fold following ceftolozane/tazobactam exposure, and the emergence of cross-resistance between ceftolozane/tazobactam and cefiderocol was demonstrated in 21% (3/14) of patients. To put these findings into context, only 6% (11/178) of all isolates studied demonstrated cefiderocol MICs ≥2 mg/L, and 73% (8/11) of these isolates were collected from patients previously treated with ceftolozane/tazobactam.

Our findings of potential cross-resistance between ceftolozane/tazobactam and cefiderocol are in agreement with prior reports.^23,25,26^ The underlying mechanisms of resistance appears to include mutations in *ampC* and TBDR genes, specifically *pirR*, a regulator of TBDR gene *piuA*. Substitutions in AmpC Ω-loop, H2 helix and regions interacting with these sites are known to change the catalytic activity of PDC, particularly among isolates with elevated ceftolozane/tazobactam MICs.^3,27–29^ During *in vitro* selection studies an L320P mutation in the R2 loop of AmpC was selected across multiple *P. aeruginosa* lineages after exposure to cefiderocol.^26^ We hypothesize that these conformational changes in PDC known to impact ceftolozane/tazobactam also affect the affinity for cefiderocol, an observation we previously demonstrated in clinical isolates of *Enterobacter cloacae* complex.^27,30^ In this regard, it is notable that isolates in the current study with the highest cefiderocol MICs (>4 mg/L) were universally resistant to ceftolozane/tazobactam and harboured mutations in *ampC*. Unlike other ceftolozane/tazobactam-resistant isolates, however, those with elevated cefiderocol MICs also encoded mutations in *pirR*, most commonly a single base-pair insertion or deletion in the same poly(dC) tract resulting in a frameshift and loss of function (Table 2, Table S3). Frameshift mutations in *pirR* were identified among isolates collected from patients treated with ceftolozane/tazobactam, and importantly not among surveillance isolates, or those collected from patients treated with ceftazidime/avibactam or imipenem/relebactam. *PirR* encodes the response regulator of the two-component PirRS system that regulates expression of TBDRs. The TBDR PiuA, and its orthologue PiuD, have been reported as the main transporters for cefiderocol as decreased expression of associated genes resulted in 128-fold increased cefiderocol MICs.^31,32^ We did not observe relevant or unique changes to *piuA* or *piuD* genes, which may be due to the fact that none of the patients in the present study were previously treated with cefiderocol. Moreover, we did not identify new mutations in *piuC,* which has been implicated in the development of cefiderocol resistance previously.^26^ Finally, it appears that decreased expression of TBDR PirA may influence cefiderocol MICs, although to a lesser extent by comparison,^31^ which aligns with the data reported herein.

Detection of cefiderocol non-susceptible *P. aeruginosa* following treatment with ceftolozane/tazobactam was first reported by Streling and colleagues^23^ in a single case. In that prior report, a patient treated with ceftolozane/tazobactam for 14 days developed treatment-emergent ceftolozane/tazobactam resistance, and a *P. aeruginosa* subpopulation demonstrated an increased cefiderocol MIC from 2 mg/L at baseline to 8 mg/L following ceftolozane/tazobactam exposure. WGS identified disruptions in both *piuD* and *pirR.* To contrast these findings, a study of 32 paired isolates collected before and after ceftolozane/tazobactam exposure in 16 patients did not identify any mutations in TBDR genes; however, at least 4-fold increases in cefiderocol MIC among post-exposure isolates were noted in 25% of cases.^25^ These cases were associated with new mutations in *ampC*, *ampD* and *mexR.* In our study, 57% (8/14) of ceftolozane/tazobactam-treated cases were associated with at least a 4-fold increase in cefiderocol MICs following exposure, and 21% (3/14) resulted in cefiderocol resistance when defined by an MIC >4 mg/L. Resistance in these cases was associated with the presence of mutations in both *ampC* and TBDR genes. The cumulative data suggest that cross-resistance between ceftolozane/tazobactam and cefiderocol is relatively uncommon, but an important phenomenon that has now been documented to varying degrees by three independent studies. Mutations in *ampC* alone are associated with ∼4-fold increased cefiderocol MICs that remain within the susceptible range (MIC ≤4 mg/L) for most isolates. Additional mutations in TBDR are likely necessary to confer resistance to cefiderocol, and seemingly arise more commonly following treatment with ceftolozane/tazobactam than other novel β-lactam/β-lactamase inhibitor combinations. These data serve as a caution for use of cefiderocol following the emergence of ceftolozane/tazobactam resistance without first confirming susceptibility.

We did not identify cross-resistance or significantly reduced susceptibility to cefiderocol among patients treated with ceftazidime/avibactam or imipenem/relebactam, which was evaluated in fewer patients overall. Among serial isolates from five patients who developed resistance to imipenem/relebactam attributed to mutations in the *mexAB-oprM* or *mexEF-oprN* efflux operons, none demonstrated a cefiderocol MIC >2 mg/L or a >4-fold increased cefiderocol MIC relative to the baseline isolate from each patient. These findings were consistent among serial isolates from patients treated with ceftazidime/avibactam where post-exposure isolates from three patients harboured mutations in *mexAB-oprM* or its repressor gene *mexR*, and each had cefiderocol MICs ≤0.25 mg/L. Our data support the hypothesis that cefiderocol is not a major substrate for efflux in *P. aeruginosa.* In fact, against engineered strains either deficient in or overexpressing *mexAB-oprM*, cefiderocol MICs were only shifted 2-fold.^32^ That said, paired clinical isolates from two patients treated with ceftolozane/tazobactam that demonstrated ≥4-fold cefiderocol MICs only showed mutations in *mexR* (A66V or L57D), but not other genes investigated.^25^ We also identified mutations at the L57 position in *mexR* (L57P and L57Q), and consistent with the previous report, cefiderocol MICs with mutations in *mexR* ranged from 0.25 to 2 mg/L. Other mutations (D89E) in *mexR* have been associated with increased cefiderocol MICs in a case report.^33^ From these data we propose that efflux is not a primary contributor to reduced cefiderocol susceptibility, but overexpression of efflux pumps may play a minor role in isolates that also overexpress *ampC*. The same is likely true of mutations in *ftsI*, which encodes PBP3, the primary inhibition target for cefiderocol. We identified *ftsI* mutations in both cefiderocol-susceptible and -resistant *P. aeruginosa* isolates without clear associations. We previously reported that *ftsI* mutations are complementary to, but not primarily responsible for, ceftolozane/tazobactam resistance,^4^ and were identified in one patient with treatment-emergent resistance to ceftazidime/avibactam in the present report.

Our data add to a growing number of reports that have identified mechanisms of cefiderocol reduced susceptibility in *P. aeruginosa* clinical isolates. Reported mechanisms broadly include mutations in iron transport systems, two component response regulators, TBDRs, *ampC* and efflux operons.^7,23,25,31,33,34^ Notably, few reports have described the emergence of cefiderocol resistance following treatment with the agent. In the CREDIBLE-CR study, 15% of isolates collected from patients treated with cefiderocol demonstrated a ≥4-fold MIC increase, including three cases involving *P. aeruginosa.*^9^ WGS of post-treatment *P. aeruginosa* isolates did not identify relevant mutations in two cases (notably *pirR* mutations were not investigated). In the third case, however, a four amino acid deletion in PDC was identified, and subsequently associated with an 8-fold cefiderocol MIC increase in isogenic strains. The data further support the role of *ampC* mutations in reduced cefiderocol susceptibility following treatment with cephalosporins.^27,30^ It remains to be seen how readily TBDR gene mutations are selected for following treatment with cefiderocol. In two previously reported cases, cefiderocol resistance emerged following treatment of XDR *P. aeruginosa* infections; however, molecular analysis of the resistant isolates was not conducted.^35,36^

In summary, we offer new and important insights for the *in vitro* activity of cefiderocol against MDR and XDR *P. aeruginosa* isolates for which cefiderocol treatment is likely to be considered. Indeed, nearly half of isolates included in this study were resistant to ceftolozane/tazobactam, ceftazidime/avibactam and/or imipenem/relebactam, including serial isolates from patients treated with these agents. The most noteworthy finding resulting from our analysis is the potential for reduced cefiderocol activity in the setting of treatment-emergent resistance to ceftolozane/tazobactam. This is particularly problematic given cross-resistance between ceftolozane/tazobactam and ceftazidime/avibactam mediated by amino acid substitutions in the catalytic centre of PDC that result in structural changes.^4,25^ Indeed, structural modifications to PDC appear to have a negative impact on all cephalosporin-based antipseudomonal agents, but fortunately are associated with collateral sensitivity to imipenem.^4,37^ Thus, treatment with imipenem/relebactam may be the most reasonable option in the setting of treatment-emergent resistance to ceftolozane/tazobactam. Susceptibility to both cefiderocol and imipenem/relebactam should be confirmed prior to treatment initiation given that clinical experience with either agent for treatment of MDR and XDR *P. aeruginosa* remains limited. It is also important to highlight that treatment-emergent resistance to ceftazidime/avibactam and imipenem/relebactam did not significantly attenuate the activity of cefiderocol. Mechanisms of resistance to these agents include increased expression of *ampC* and efflux pumps that minimally impact cefiderocol *in vitro* activity. Overall, this is consistent with the broader potency of cefiderocol against *P. aeruginosa.* Finally, it should be noted that further validation is needed to confirm mechanisms of reduced cefiderocol activity, and in particular the association of adaptive TBDR gene mutations following treatment with ceftolozane/tazobactam. Nevertheless, the *in vitro* activity of cefiderocol against this challenging set of *P. aeruginosa* isolates supports routine cefiderocol testing for clinical isolates where antibiotic options are limited.

## Supplementary Material

dlad107_Supplementary_Data

## Supplementary data

Tables S1 to S3 are available as Supplementary data at *JAC-AMR* Online.
